# Development and Performance Analysis of Pneumatic Soft-Bodied Bionic Actuator

**DOI:** 10.1155/2021/6623059

**Published:** 2021-02-17

**Authors:** Wenchuan Zhao, Yu Zhang, Ning Wang

**Affiliations:** School of Mechanical Engineering, Shenyang University of Technology, Shenyang 110870, China

## Abstract

The design of a pneumatic soft-bodied bionic actuator derives from the structural characteristics and motion mechanism of biological muscles, combined with the nonlinear hyperelasticity of silica gel, which can improve the mobility and environmental adaptability of soft-bodied bionic robots. Based on Yeoh's second-order constitutive model of silica gel, the deformation analysis model of the actuator is established, and the rationality of the structure design and motion forms of the actuator and the accuracy of the deformation analysis model are verified by using the numerical simulation algorithm. According to the physical model of the pneumatic soft-bodied bionic actuator, the motion and dynamic characteristics of the actuator are tested and analyzed, the curves of motion and dynamic characteristics of the actuator are obtained, and the empirical formula of the bending angle and driving torque of the actuator is fitted out. The results show that the deformation analysis model and numerical simulation method are accurate, and the pneumatic soft-bodied bionic actuator is feasible and effective, which can provide a design method and reference basis for the research and implementation of soft-bodied bionic robot actuator.

## 1. Introduction

The traditional robot is composed of a large number of rigid parts, which face the disadvantages of low human-computer interaction security, poor adaptability in unstructured environment, low driving efficiency, and high maintenance cost and has gradually failed to meet the actual needs. With the thriving of bionic robot technology, material technology, and rapid prototyping technology, the soft-bodied bionic robot with high flexibility, safety, and efficiency has become a new direction in the field of robot development [1–4]. Among them, the actuator is the main component in realizing the motion function of the soft-bodied bionic robot. Therefore, in order to improve the maneuverability of the soft-bodied bionic robot, researchers have developed different types of actuators [5, 6].

Najem and Leo [7] from the Virginia Institute of Technology have developed an IPMC actuator controlled by electrical signals. Through the bending deformation of the IPMC actuator, the soft-bodied bionic jellyfish robot can achieve contraction and expansion. Shepherd et al. [8] from the Harvard University have developed an actuator driven by chemical explosion. Through the explosion impact of chemical fuel, the soft-bodied bionic robot can realize the jumping motion. Villanueva et al. [9] from the University of Virginia have developed an actuator driven by an electric motor, which enables the soft-bodied bionic robot to realize underwater propulsion and swimming posture adjustment. Renda et al. [10] from the University of Khalifa have developed an actuator driven by cables, through imitating the motion mechanism of biological arms; the soft-bodied bionic robot can realize amphibious mobility. Researchers [11] from BEAR Laboratory of the University of Maine have developed a voltage-controlled composite material actuator made from ionic polymers and nanometal. Through the bending deformation of the material body, the soft-bodied bionic robot can achieve the required motion forms. Wehner et al. [12] from the Weiss Institute of Bioengineering in Harvard University have developed an actuator based on platinum catalytic reaction, which enables the soft-bodied bionic octopus robot to realize position and orientation transformation. Rodrigue et al. [13] from the Seoul National University have developed an actuator driven by a memory alloy material. By inserting two memory alloy wires with opposite eccentric angles inside the polymer, the soft-bodied bionic robot is capable of performing related tasks. Wang et al. [14] from the Tsinghua University have developed an actuator driven by adding a certain amount of ethanol to the liquid metal composite materials. Based on the principle that ethanol volatilizes with the change of temperature, the soft-bodied bionic octopus robot can achieve the change of posture. Musta et al. [15] from the Tartu University have developed an actuator driven by the electrical excitation process of ion penetration, and the driving process is cyclic reversible. Yichuan et al. [16] from the Shenzhen University of Geneva Raklev have developed an actuator driven by the piezoelectric chip structure under the action of AC voltage. Based on the principle of piezoelectric contraction, the position and pose of the soft-bodied bionic robot can be adjusted.

The driving methods of the above soft-bodied bionic robot actuators can be classified as chemical driven, intelligent material driven, and electric driven. These driving methods are mainly faced with low driving efficiency, poor stability, and limited pose transformation. Therefore, pneumatic and hydraulic driven soft-bodied actuators and the corresponding soft-bodied bionic robots are gaining favor, because of their outstanding characteristics of simple driving methods, high driving efficiency, strong environmental adaptability, and smooth posture transformation.

The soft-bodied bionic underwater robot [17–19] driven by pneumatic and hydraulic actuators can fully imitate the structural characteristics and motion mechanism of underwater organisms. Through the study of its swing frequency and amplitude, the soft-bodied robot can achieve high driving efficiency and stability. The soft-bodied bionic crawling robot driven by pneumatic and hydraulic actuators [20–22] can fully imitate the structural characteristics and motion mechanism of worms. Through the study of the relationship between friction and driving force, the soft-bodied robot can realize relevant operations in planes, pipeline, and other environments. The snake-like soft-bodied bionic robot driven by pneumatic and hydraulic actuators [23, 24] can fully simulate the structural characteristics and motion mechanism of snake-like organisms. Through its zigzag form of pose transformation, the soft-bodied robot can work flexibly in narrow spaces. The wheel-walking soft-bodied bionic robot driven by pneumatic and hydraulic actuators [25], based on its wheel-walking structural characteristics, can achieve rapid maneuvering through periodic bending changes of the body structure. The pneumatic and hydraulic driven actuators of soft-bodied bionic joint [26] can boost its power output by adjusting the output of the driving torque. The soft-bodied bionic robotic arm driven by pneumatic and hydraulic actuators [27, 28] controls the internal pressure of the driving cavity structure through the pressure proportional valve, so that it can realize flexible operation and has the function of elongation. The soft-bodied bionic robotic manipulator driven by pneumatic and hydraulic actuators [29, 30] can guarantee the grasping and releasing of single or multiple target objects without doing any damage to them. Pneumatic and hydraulic driven soft-bodied bionic basic execution unit [31–33] can realize the basic driving modes and purposes according to the driving cavity of different types of designed structure.

Although the abovementioned pneumatic and hydraulic driven soft-bodied bionic actuators can meet the corresponding design requirements, there exist drawbacks including poor environmental adaptability, single motion form, and lack of systematic research methods. Besides, most of the researches are limited to the type of entirely soft-bodied or filament+soft-bodied, which calls for the improvement of structure strength, execution strength, and resilience of the pneumatic soft-bodied bionic actuators, making it difficult to lay a good foundation for further application. Therefore, this paper deriving from the nature of structural characteristics and motion mechanism of biological muscles, and making full use of the advantages of the silica gel material, the structure design, mechanical model, numerical simulation algorithm verification, physical model preparation, experimental test analysis of the pneumatic soft-bodied bionic actuator are carried out while simplifying the structure of the bionic body, so as to greatly improve its environmental adaptability and motion performance and broaden its application field. Besides, the pneumatic network devices of the actuator are independent of each other, which can not only effectively avoid mutual coupling interference but also improve the motion performance. Therefore, this paper can provide a reference for the research of pneumatic soft-bodied bionic robot actuator, especially through the theoretical modeling of the soft-bodied actuator embedded with pressure spring structure; it can also provide a new idea for the theoretical research of the soft-bodied actuator. It should be noted that the actuator can be used in mobile robots and manipulating robots. For the mobile robot equipped with the actuator, in the underwater environment, it can simulate the motion form of the torso undulating type fishtail and the torso swinging type fishtail, so that the robots can swim and turn the rudder quickly; in the land environment, it can simulate the motion mechanism of the inchworm, so that the robots can crawl and change direction. For the manipulative robots equipped with this actuator, different numerical pressure can be applied to the middle, left, and right actuator units, so that the manipulator can complete certain work requirements.

## 2. Structure Design and Motion Forms Analysis

### 2.1. Structure Design

**Figure 1:**
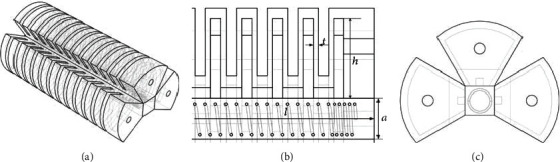
Structure diagram of pneumatic soft-bodied bionic actuator. (a) Overall structure of actuator. (b) Partial view of actuator cross section. (c) End view of actuator.

The pneumatic soft-bodied bionic actuator is shown in Figure 1. Its structure is mainly composed of three pneumatic network devices, a nonretractable column structure, and a compression spring structure. Each actuating unit is composed of a pneumatic network device, a nonretractable column structure, and a compression spring structure, and each pneumatic network device is composed of 13 driving cavity structures. Besides, in order to enhance the structural strength, execution force, and motion restoring force of the actuator, the compression spring structure is embedded in the inner part of the nonretractable column.

The main structural parameters of the actuator are shown in Table 1. It should be noted that in order to ensure the interaction between the driving cavity structures of actuator, the other walls of the driving cavity structures are required to be thicker than the expansion walls.

### 2.2. Analysis of Motion Forms

**Figure 2:**
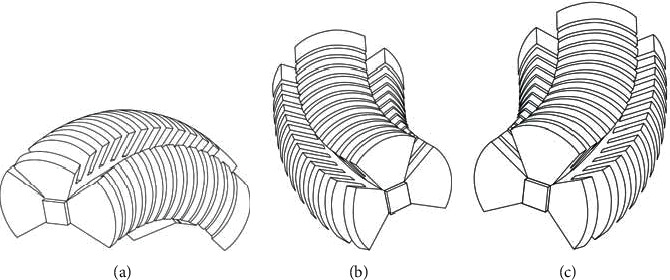
Periodic motion process diagram of pneumatic soft-bodied bionic actuator. (a) Middle part bending, (b) Left side bending. (c) Right side bending.

The motion process of the pneumatic soft-bodied bionic actuator can be divided into two parts: the middle part periodic bending and the left and right sides periodic bending. In the middle part periodic bending motion stage, when the air pump inflates the inner part of the middle part actuating unit of the actuator, driving cavity structures to interact with each other, resulting in the expansion and bending deformation of the middle part of the actuator, as shown in Figure 2(a). In the left side periodic bending motion stage, when the air pump inflates the left side actuating unit of the actuator, the left side of the actuator will expand and bend due to the interaction of the driving cavity structures, as shown in Figure 2(b). In the right side periodic bending motion stage, when the air pump inflates into the right side actuating unit of the actuator, the right side of the actuator will expand and bend due to the interaction of the driving cavity structures, as shown in Figure 2(c).

## 3. Mechanical Model Analysis

### 3.1. Constitutive Model of Silica Gel Material

The pneumatic soft-bodied bionic actuator is mainly composed of silica gel material, which has the characteristics of hyperelasticity and the capability of large deformation. There exists a highly nonlinear relationship between its strain and stress. In order to ensure the reliability of the research results, the phenomenological theory is used to describe its mechanical properties. The common constitutive models of silica gel materials are the Mooney Rivlin model, Ogden model, Yeoh model, etc. [34–38]. Among them, the Yeoh model has good adaptability in the range of 300% deformation, which is the first choice for analyzing the deformation of silica gel.

Based on the stress-strain theory, the constitutive relation of silica gel material is established, and it is assumed that the silica gel material is isotropic and incompressible. Equation (1) of the strain energy function is as follows:

(1) $$\begin{array}{c} \left\{\begin{array}{c} W=W\left(I_{1},I_{2},I_{3}\right),\\ I_{1}=\lambda_{1}^{2}+\lambda_{2}^{2}+\lambda_{3}^{2},\\ I_{2}=\lambda_{1}^{2}\lambda_{2}^{2}+\lambda_{2}^{2}\lambda_{3}^{2}+\lambda_{1}^{2}\lambda_{3}^{2},\\ I_{3}=\lambda_{1}^{2}\lambda_{2}^{2}\lambda_{3}^{2}, \end{array}\right. \end{array}$$

where *I*_1_, *I*_2_, and *I*_3_ are the invariants of the deformation tensor; *λ*_1_, *λ*_2_, and *λ*_3_ are the main elongation ratio in three directions of space; and *W* is the strain energy function.

According to the isotropy and incompressibility of the materials, Equation (2) is listed as follows:

(2) $$\begin{array}{c} I_{3}=\lambda_{1}^{2}\lambda_{2}^{2}\lambda_{3}^{2}=1. \end{array}$$

During the expansion of the driving cavity structure, the expansion wall will not only elongate but also will become thinner. So, it is necessary to assume that the expansion wall does not deform in the width direction, that is, *λ*_3_ = 1. Therefore, according to the incompressibility of the material constitutive model, Equation (3) is listed as follows:

(3) $$\begin{array}{c} \lambda_{2}^{2}=\frac{1}{\lambda_{1}^{2}}. \end{array}$$

The deformation tensor can be expressed as in

(4) $$\begin{array}{c} I_{1}=I_{2}=\lambda_{1}^{2}+\frac{1}{\lambda_{1}^{2}}+1. \end{array}$$

Based on the most typical second-order parameters form of the Yeoh model, Equation (5) of the strain energy density function model is expressed as

(5) $$\begin{array}{c} W=C_{10}\left(I_{1}-3\right)+C_{20}{\left(I_{1}-3\right)}^{2}=C_{10}{\left(\lambda_{1}-\frac{1}{\lambda_{1}}\right)}^{2}+C_{20}{\left(\lambda_{1}-\frac{1}{\lambda_{1}}\right)}^{4}, \end{array}$$

where *C*_*ij*_ is the dimensionless relationship constant, *C*_10_ = 0.09, *C*_20_ = 0.02.

Combining Equation (1) to Equation (5), the expression of principal stress can be derived from the Piola-Kirchhoff stress and Cauchy-Green strain relationship, and thus, Equation (6) is listed as follows:

(6) $$\begin{array}{c} \sigma_{ij}=\frac{2}{\lambda_{1}}\left(\lambda_{1}^{2}‐\frac{1}{\lambda_{1}^{2}\lambda_{2}^{2}}\right)\left(\frac{\partial W}{\partial I_{1}}+\lambda_{2}^{2}\frac{\partial W}{\partial I_{2}}\right). \end{array}$$

Substituting formula Equation (3) to Equation (5) into Equation (6), the relationship between stress and the main elongation ratio, i.e., Equation (7) is listed as follows:

(7) $$\begin{array}{c} \sigma =\frac{\lambda_{1}^{4}‐1}{\lambda_{1}^{3}}\left(2C_{1}+4C_{2}{\left(\lambda_{1}-\frac{1}{\lambda_{1}}\right)}^{2}\right). \end{array}$$

### 3.2. Establishment of Mechanical Model

**Figure 3:**
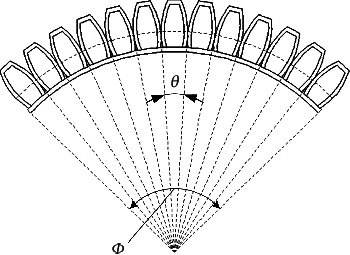
Expansion equivalent diagram.

It is difficult to model the pneumatic soft-bodied bionic actuator, so it is necessary to simplify it properly in the nonlinear mechanical analysis. Since the actuator is composed of three identical actuating units arranged at equal angles, and driven in different time sequences according to the specific operation requirements, the equivalent bending deformation diagram can be obtained by taking the inflation of a single actuating unit as an example for theoretical modeling, as shown in Figure 3. Among them, the overall bending angle is indicated by *Φ*, and the bending angle of the single driving cavity structure is indicated by *θ*.

According to Figure 3, the overall bending angle is the result of the bending superposition of each driving cavity structure, and the relationship between the overall bending angle *Φ* and *θ* can be expressed as in

(8) $$\begin{array}{c} \Phi =13\theta . \end{array}$$

**Figure 4:**
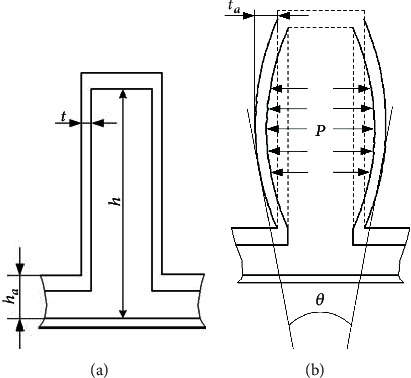
Equivalent diagram of a single driving cavity structure. (a) Initial state. (b) Deformation state.

Then, the structure of a single driving cavity is researched, and the equivalent model of the initial state and expansion deformation state is obtained, as shown in Figure 4.

When the expansion wall expands outwards under the action of inflation pressure *P*, the lateral distance of expansion is *t*_*a*_.

The bending angle *θ* of a single driving cavity structure can be expressed as in

(9) $$\begin{array}{c} \theta =2\arctan\left(\frac{t_{a}}{\left(\left(h‐h_{a}\right)/2\right)+h_{a}}\right). \end{array}$$

**Figure 5:**
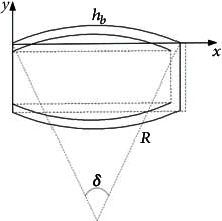
Coordinate system of a single driving cavity structure.

Then, take the bottom end of the expansion wall of the driving cavity structure as the coordinate origin, and thus, the coordinate system is established, as shown in Figure 5. Among them, the center angle formed by the bulge of the expansion wall is *δ*, the radius of the arc is *R*, the height of the expansion wall elongates from *h* to the arc length *h*_*b*_ after inflation, and the center coordinate is (*x*_0_, *y*_0_).

Then, the relation between *h*_*b*_ and *h* is expressed as in

(10) $$\begin{array}{c} h_{\text{b}}=\lambda_{1}h. \end{array}$$

In the established coordinate system, the arc of the expansion wall can be expressed as in

(11) $$\begin{array}{c} y=\sqrt{R^{2}-{\left(x-x_{0}\right)}^{2}}+y_{0}, \end{array}$$

Then, according to the geometric relationship, Equation (12) is obtained as follows:

(12) $$\begin{array}{c} \left\{\begin{array}{c} x_{0}=\frac{h‐h_{a}}{2},\\ y_{0}=-R\cos\left(\frac{\delta }{2}\right),\\ R=\frac{h‐h_{a}}{2\cdot \sin\left(\delta /2\right)}. \end{array}\right. \end{array}$$

It can be expressed as in

(13) $$\begin{array}{c} y\left(\frac{h‐h_{a}}{2}\right)=\sqrt{R^{2}-{\left(\frac{h‐h_{a}}{2}-x_{0}\right)}^{2}}+y_{0}=y\left(\delta \right). \end{array}$$

Combining Equation (8) to Equation (13), the relationship between *Φ* and *δ* can be obtained as in

(14) $$\begin{array}{c} \Phi =\Phi \left(\delta \right). \end{array}$$

Through the relationship between the inflation pressure *P* and the center angle *δ* of the expansion wall bulge, the relationship between the inflation pressure *P* and the overall bending angle *Φ* is obtained. Then, assuming that the inflation pressure increases from 0 to *P*, and the deformation of each point on the expansion wall increases linearly to*y*(*x*), the work done by the pressure at each point on the side wall to the side wall can be expressed as in

(15) $$\begin{array}{c} U=26\int_{0}^{h_{b}}P\cdot y\left(x\right)dx=26P\int_{0}^{\lambda_{1}h}\left(\sqrt{R^{2}-{\left(x-x_{0}\right)}^{2}}+y_{0}\right)\text{d}x=U\left(P,\delta \right). \end{array}$$

The main elongation *λ*_1_ is expressed by *δ*, as in

(16) $$\begin{array}{c} \lambda_{1}=\frac{\delta }{2\cdot \sin\left(\delta /2\right)}. \end{array}$$

Taking Equation (3) to Equation (5) into consideration, the function of strain energy density can be expressed as a function of *δ*, as in

(17) $$\begin{array}{c} W=W\left(\delta \right). \end{array}$$

The deformation energy of the expansion wall after deformation can be expressed as in

(18) $$\begin{array}{c} V=26t\cdot \left(h‐h_{a}\right)\cdot W\left(\delta \right)=V\left(\delta \right). \end{array}$$

The deformation energy of the nonretractable column corresponding to the deformation of the expansion wall can be expressed as in

(19) $$\begin{array}{c} E=l\cdot a\cdot W\left(\delta \right)=E\left(\delta \right). \end{array}$$

The work done by the compression spring corresponding to the deformation of the expansion wall is

(20) $$\begin{array}{c} E_{1}\left(\delta \right)=\frac{1}{2a}{kl}^{2}, \end{array}$$

where *k* is the elasticity coefficient, the value is 1.1.

According to the law of conservation of energy, the work done at each point on the expansion wall is equal to the deformation energy of the side wall of the driving cavity structure, and therefore, Equation (21) is listed as follows:

(21) $$\begin{array}{c} V\left(\delta \right)=U\left(P,\delta \right)‐E\left(\delta \right)‐E_{1}\left(\delta \right). \end{array}$$

Combining Equation (14) and Equation (21) can further derive the function expression between the bending angle *Φ* and the inflation pressure *P*; this paper does not need to elaborate but uses Equation (22) to express it.

(22) $$\begin{array}{c} \Phi =\Phi \left(P\right). \end{array}$$

## 4. Numerical Simulation Algorithm Verification and Physical Model Preparation

### 4.1. Verification of Numerical Simulation Algorithm

**Figure 6:**
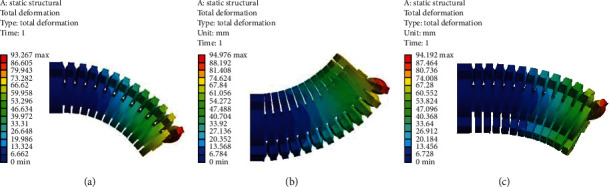
Pressurize expansion and bending of pneumatic soft-bodied bionic actuator. (a) Middle part actuating unit. (b) Left side actuating unit. (c) Right side actuating unit.

Based on Yeoh's second-order constitutive model of silica gel material for the pneumatic soft-bodied bionic actuator, the rationality of structural design and motion forms and the accuracy of the theoretical model of deformation analysis are verified by the numerical simulation algorithm [39–41]. The calculation results obtained from the numerical simulation algorithm are shown in Figure 6.

According to Figure 6, the deformation of the pneumatic soft-bodied bionic actuator conforms to the actual deformation of the silica gel material after inflation pressure and can meet the analysis results of motion forms. Therefore, the deformation change law of the cloud diagram can verify the rationality of the structure design and motion forms of the actuator.

It should be noted that the theoretical model of deformation analysis is based on the simplified equivalent bending deformation of the actuator, which is more similar to the deformation and bending of the middle part actuating unit of the actuator. Therefore, the theoretical model is verified by the numerical simulation results of deformation and bending of the middle part actuating unit of the actuator, and the numerical simulation algorithm calculation results under different driving pressures from 10 kPa to 80 kPa are obtained, as shown in Table 2.

**Figure 7:**
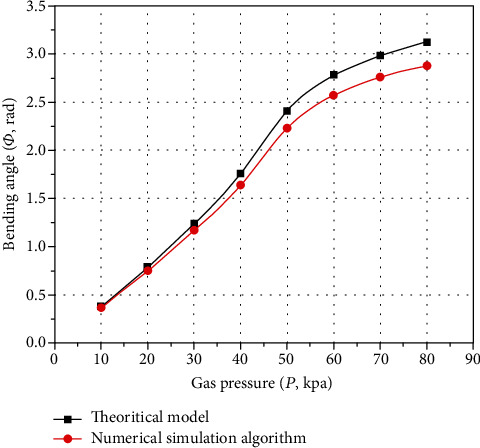
Error analysis of theoretical deformation analysis model.

By comparing the calculation results in Table 2 with the theoretical model of deformation analysis, as shown in Figure 7.

According to Figure 7, the calculation results of the numerical simulation algorithm and the theoretical model have the same curve change trend. Although the absolute error of the numerical simulation calculation results and the theoretical model gradually increases with the increase of the inflation pressure, the overall absolute errors change is still relatively accurate. The maximum absolute error is 0.24 rad, and the maximum relative error is 7.69%. Therefore, the data measurement is relatively reliable and the theoretical model of deformation analysis has certain accuracy. Besides, it should be noted that the main reason for the errors is that the theoretical model only considers the expansion and bending changes of a single actuating unit.

### 4.2. Physical Model Preparation

The body structure of the pneumatic soft-bodied bionic actuator needs to have certain strength and flexibility. Deficiency in hardness results in overly soft structure, and consequently insufficient strength, and poor execution force, On the other hand, excessive hardness results in brittleness, making the structure easy to fracture [42–44]. Therefore, the room temperature-vulcanized silica gel with *shore A20* hardness was selected.

#### 4.2.1. Material Preparation

The materials required for the pneumatic soft-bodied bionic actuator are shown in Table 3.

#### 4.2.2. Mould Manufacture

**Figure 8:**
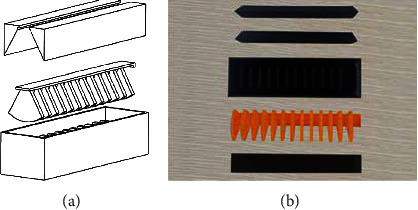
Mould structure. (a) Mould design of pneumatic network device. (b) Mould physical model.

According to the specific structural features of the actuator, based on 3D printing technology, the solid model of the mould is obtained [45–47], as shown in Figure 8. Among them, in order to demould smoothly, based on the lost wax casting technology, the mould core which forms the internal structure of the pneumatic network device is printed by paraffin wax material.

#### 4.2.3. Preparation of Physical Objects

**Figure 9:**
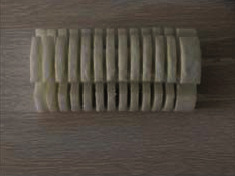
Physical model of pneumatic soft-bodied bionic actuator.

Silica gel pouring, static forming, and demoulding are carried out based on the compound modeling process, and the core of the mould is melted by high-temperature melting technology, and the silica gel bonding technology is used for bonding [48]. Finally, the physical model of the pneumatic soft-bodied bionic actuator is completed, see Figure 9.

## 5. Experimental Test and Analysis

**Figure 10:**
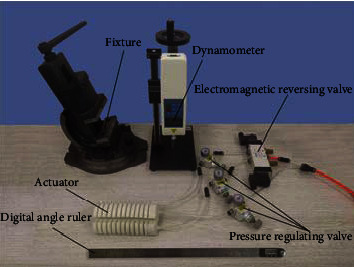
Experimental test platform.

Through experimental testing and analysis, the exploration of the influence of different inflation pressure conditions on the motion and dynamic characteristics of the pneumatic soft-bodied bionic actuator lays the foundation for further application [49]. The main devices of the experiment include pneumatic soft-bodied bionic actuator, pressure regulating valves, electromagnetic reversing valve, fixture, digital angle ruler, and dynamometer. These devices are shown in Figure 10.

Among them, the inflatable end of the actuator is fixed and then the pressure from 10 kPa to 80 kPa is filled into the actuating unit by the air pressure pump.

### 5.1. Kinematic Characteristics Analysis of Pneumatic Soft-Bodied Bionic Actuator

#### 5.1.1. Motion Frequency Analysis

**Figure 11:**
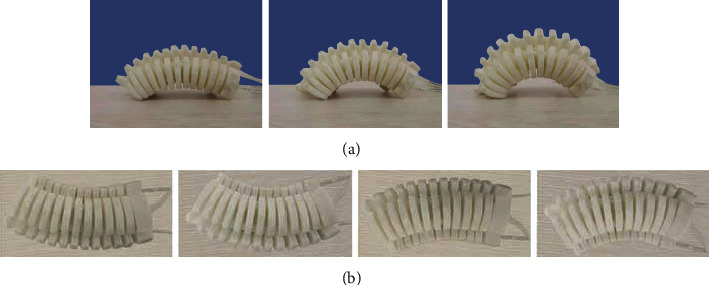
Motion process of pneumatic soft-bodied bionic actuator. (a) Middle part bending motion of actuator. (b) Left and right side bending motion of actuator.

On the premise of ensuring the same switching response time of the electromagnetic reversing valve, the motion frequency of the pneumatic soft-bodied bionic actuator is collected by the video camera. In the middle part periodic bending motion stage of actuator, the inflation pressure is decided, and deflation is conducted immediately after the completion of inflation; the experiment continues until the actuator reaches a straight state, so as to determine the whole cycle time, and to complete the research on the inflation and deflation of the middle part periodic bending motion of the actuator, as shown in Figure 11(a). In the left and right side periodic bending motion stage of actuator, first the inflation pressure of actuator on one side is determined, and after inflation completes, it is deflated immediately, while the other side of actuator inflates. After inflation, the actuator deflates immediately until the end of deflation and the actuator reaches a straight state, as shown in Figure 11(b).

**Figure 12:**
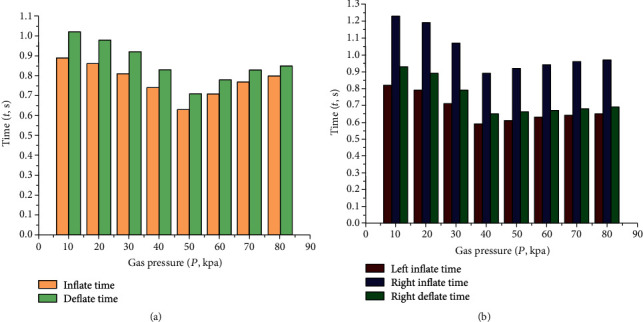
Motion cycle sequence diagrams of pneumatic soft-bodied bionic actuator. (a) Middle part bending motion cycle of actuator. (b) Left and right side bending motion cycle of actuator.

After data acquisition, the sequence diagram of the cycle of inflation and deflation of actuator is obtained, as shown in Figure 12.

According to Figure 12, the middle part periodic bending time of the pneumatic soft-bodied bionic actuator is shorter than that of the left and right sides, because it only does one-way bending motion. Besides, the unilateral motion time of the middle part periodic bending motion is longer than that of the left and right side periodic bending motion, which is caused by the larger amplitude of the middle part periodic bending motion compared with that of the left and right side periodic bending motion under the same inflation pressure. It should be noted that the periodic bending amplitude of the middle part increases as the inflation pressure increases, while the recovery force of the compression spring structure is also increasing, which makes the recovery time of the actuator structure gradually approximate the time of inflation bending gradually.

**Figure 13:**
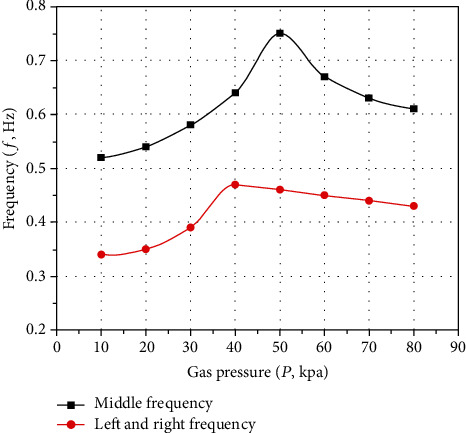
*P* − *f* characteristic curves of pneumatic soft-bodied bionic actuator.

Then, according to the periodic sequence of actuator, the *P* − *f* characteristic curves of inflation pressure can be obtained, as shown in Figure 13.

Figure 13 shows that the motion frequency of the pneumatic soft-bodied bionic actuator first increases and then decreases with the increase of inflation pressure, which is caused by the increase of its motion amplitude. When the motion amplitude is relatively small, the motion frequency increases with the increase of inflation pressure, and when the motion amplitude is relatively large, the motion frequency decreases with the increase of inflation pressure. Among them, the motion frequency of the middle part periodic bending is higher than that of the left and right side periodic bending.

#### 5.1.2. Bending Angle Analysis

**Figure 14:**
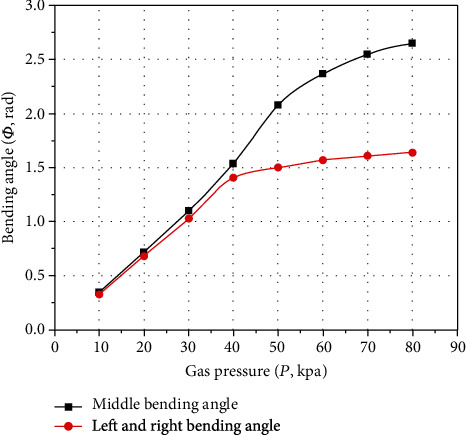
*P* − *Φ* characteristic curves of pneumatic soft-bodied bionic actuator.

The *P* − *Φ* characteristic curves between the inflation pressure and the bending angles of the actuator can be obtained by collecting the bending angles of the pneumatic soft-bodied bionic actuator with the digital angle ruler, as shown in Figure 14.

Figure 14 shows that the maximum bending angles produced by the periodic bending motion on the middle part of the pneumatic soft-bodied bionic actuator and the periodic bending motion on the left and right sides are 2.65 rad and 1.64 rad, respectively. Within this range, the periodic bending motion of middle part and the periodic bending motion of left and right sides can, respectively, guarantee the operation in the best state. Among them, the *P* − *Φ* characteristic curves change gradually from the initially linear to later nonlinear, due to the expansion of the actuating unit reaching a certain extent, and the large reverse bending motion produced by the nonretractable column of silica gel body and the compression spring structure. The bending angle of the left and right side periodic bending motion is smaller than that of the middle part periodic bending motion, because it also bears the reverse bending motion produced by the compressed side when bending. It should be noted that if the inflatable pressure continues to increase, although the bending angle of the actuator will continue to increase slowly, its motion characteristics will become worse, the execution ability will be weakened, and the operation effect will be affected to a certain extent.

According to the *P* − *Φ* characteristic curves, an empirical equation (23) can be obtained, which can reflect the change of its characteristics.

(23) $$\begin{array}{c} f\left(x\right)=p_{1}\cdot x^{4}+p_{2}\cdot x^{3}+p_{3}\cdot x^{2}+p_{4}\cdot x+p_{5}. \end{array}$$

The specific scaling coefficients are shown in Table 4.

**Figure 15:**
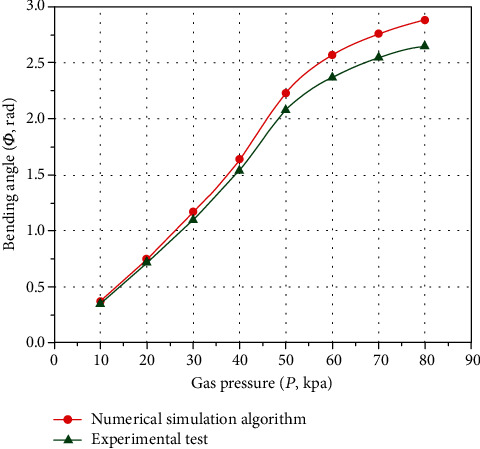
Errors analysis of numerical simulation deformation model.

The *P* − *Φ* characteristic curves obtained from the experimental test and the calculation results of the numerical simulation algorithm undergo the accuracy analysis of errors, and the results are shown in Figure 15.

According to Figure 15, the calculation results of the experimental test and the numerical simulation calculation results have the same curve change trend. Although the absolute errors of the experimental test and the numerical simulation calculation result gradually increase with the increase of the inflation pressure, the overall absolute errors change is still relatively accurate. The maximum absolute error is 0.23 rad, and the maximum relative error is 7.99%. Therefore, the data measurement is relatively reliable, and the numerical simulation calculation results and the theoretical model of deformation analysis have certain accuracy. Besides, it should be noted that the main causes of experimental test errors are measurement errors, manufacturing errors, experimental instrument errors, etc.

### 5.2. Analysis of Dynamic Characteristics of Pneumatic Soft-Bodied Bionic Actuator

**Figure 16:**
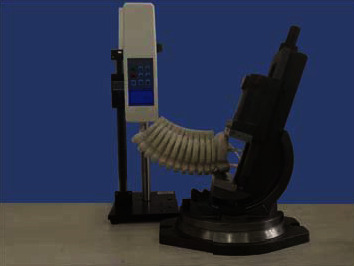
Driving torque measure experiment of pneumatic soft-bodied bionic actuator.

When analyzing the dynamic characteristics of pneumatic soft-bodied bionic actuator, the driving torque is collected by dynamometer, as shown in Figure 16.

#### 5.2.1. Dynamic Characteristic Analysis of Variable Bending Angle

**Figure 17:**
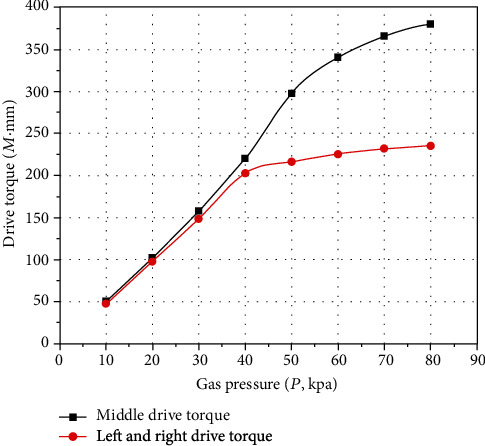
*P*‐*M* characteristic curves of pneumatic soft-bodied bionic actuator for variable angle.

The bending angle of the actuator is variable; the *P* − *M* characteristic curves of inflation pressure and driving torque are obtained, as shown in Figure 17.

Figure 17 shows that the driving torque values of the left and right side periodic bending of the pneumatic soft-bodied bionic actuator is relatively lower compared with that of the middle part periodic bending, which is caused by the counter torque produced by the compressed side cavity structure. It should be noted that the variation trend of the *P* − *M* characteristic curves is similar to that of the *P* − *Φ* characteristic curves, which indicates that the driving torque is closely related to the bending angle. In addition, with the increase of inflation pressure, the nonlinear change is partly due to the extreme deformation of the actuator body structure, which leads to the change of its stress form.

The *P* − *M* characteristic curves are expressed by the empirical equation (23), and the specific scale coefficients are shown in Table 5.

#### 5.2.2. Dynamic Characteristic Analysis of Fixed Bending Angle

**Figure 18:**
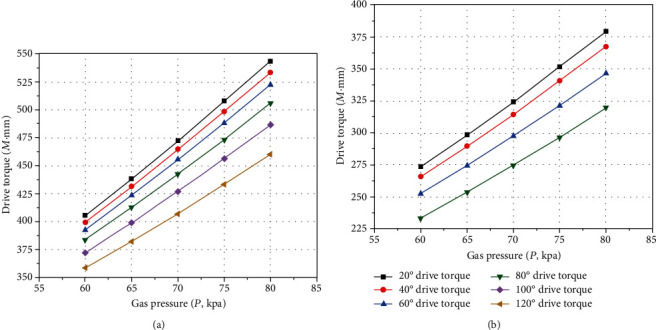
The *P* − *M* characteristic curves of pneumatic soft-bodied bionic actuator for fixed angle. (a) Middle part driving torque. (b) Left and right side driving torque.

The bending angle of the actuator is fixed; the *P* − *M* characteristic curves of inflation pressure and driving torque are obtained, as shown in Figure 18.

According to Figure 18, the middle part driving torques of the actuator and the left and right sides both increase linearly with the increase of the inflation pressure. It should be noted that the middle part driving torque of the actuator is larger than that of the left and right sides, because the left and right sides need to bear the reverse bending motion produced by the compressed side when bending; this phenomenon can correspond to the above test results.

## 6. Conclusions

This paper designs a kind of pneumatic soft-bodied bionic actuator, which is suitable for robot carriers of underwater swimming and land crawling. It has the advantages of simple structure, strong environmental adaptability, high driving efficiency, etc.

1. Based on Yeoh's second-order constitutive model of the silica gel material, the deformation analysis model of the actuator is established, and the relationship between the bending angle of the actuator and the inflation pressure is determined, which can provide a theoretical reference for the structural design and deformation analysis of the actuator of the soft-bodied bionic robot
2. Using the numerical simulation algorithm, the rationality of the actuator structure design and motion form is verified, and the accuracy of the theoretical model of deformation analysis is determined, and the accuracy of the deformation analysis theoretical model is determined through the error accuracy analysis results of the numerical simulation algorithm and experimental test. Among them, the maximum absolute error and relative error between the calculation results of the numerical simulation algorithm and theoretical model are 0.24 radian and 7.69%, and the maximum absolute error and relative error of the experimental test and calculation results of numerical simulation algorithm are 0.23 radian and 7.99%
3. The physical model of the actuator is verified and analyzed via the experimental test platform. According to the values of inflation pressure filled into the actuating unit, the motion and dynamic output in a certain range are matched, and the characteristic curves of the motion frequency, bending angle and driving torque of the actuator, and the correlative empirical formulas are obtained. The accuracy of the deformation analysis model and the numerical simulation algorithm is verified, and the actuator proves to be reasonable and feasible, which can be effectively used as the actuator of the soft-bodied bionic robot

## Figures and Tables

**Table 1:** Main structural parameters of actuator.

No.	Size	Numerical values
1	Driving cavity structure internal height *h*	35 mm
2	Driving cavity structure expansion wall thickness*t*	2 mm
3	Driving cavity structure expansion wall bottom height *h*_*a*_	8 mm
4	Nonretractable column structure thickness *a*	16 mm
5	Nonretractable column structure length *l*	160 mm
6	Driving cavity structure number *N*	13

**Table 2:** Numerical simulation algorithm calculation results.

Driving pressure (*P*, kPa)	10	20	30	40	50	60	70	80
Bending angle (*Φ*, rad)	0.37	0.75	1.17	1.64	2.23	2.57	2.76	2.88

**Table 3:** Material preparation.

Preparation objects	Material details
Pneumatic network device	601 room temperature-vulcanized silica gel
Nonretractable column	601 room temperature-vulcanized silica gel
Compression spring	Quenched 65 Mn
Mould	PLA and paraffin wax

**Table 4:** Empirical formula coefficients of *P*‐*Φ* characteristic curves.

Empirical formula coefficients	Middle part bending angle	Left and right side bending angle
*p*_1_	6.25*E* − 8	2.28*E* − 7
*p*_2_	−2.02*E* − 5	−3.99*E* − 5
*p*_3_	1.64*E* − 3	1.95*E* − 3
*p*_4_	−3.94*E* − 3	1.27*E* − 3
*p*_5_	2.57*E* − 1	1.58*E* − 1

**Table 5:** Empirical formula coefficients of *P* − *M* characteristic curves.

Empirical formula coefficients	Middle part bending angle	Left and right side bending angle
*p*_1_	9.36*E* − 6	3.26*E* − 5
*p*_2_	−2.99*E* − 3	−5.72*E* − 3
*p*_3_	2.43*E* − 1	2.79*E* − 1
*p*_4_	−7.80*E* − 1	−2.02*E* − 1
*p*_5_	38.29	22.7

## Data Availability

All data generated or analyzed used to support the findings of this study are included within the article.
